# Reporting Misconduct of a Coworker to Protect a Patient: A Comparison between Experienced Nurses and Nursing Students

**DOI:** 10.1155/2014/413926

**Published:** 2014-10-14

**Authors:** Abraham Mansbach, Talma Kushnir, Hana Ziedenberg, Yaacov G. Bachner

**Affiliations:** ^1^Department of Philosophy, Ben-Gurion University of the Negev, P.O. Box 653, 84105 Beer Sheva, Israel; ^2^Department of Public Health, Ben-Gurion University of the Negev, P.O. Box 653, 84105 Beer Sheva, Israel; ^3^Department of Nursing, Ben-Gurion University of the Negev, P.O. Box 653, 84105 Beer Sheva, Israel

## Abstract

*Purpose*. Whistleblowing is the reporting of illegal, immoral, or illegitimate practices to persons or organizations that may affect the action. The current study compares experienced nurses to nursing students regarding their willingness to blow the whistle to protect a patient's interests. *Methods*. 165 participants were divided into two groups: 82 undergraduate nursing students and 83 experienced nurses. Participants responded to two vignettes that described a colleague's and a manager's misconduct at work. *Results*. The nursing students perceived the severity of the misconduct significantly lower compared to the experienced nurses. The nursing students also ranked the internal and external whistleblowing indices higher than the nurses, but the differences did not reach statistical significance. For each of the examined internal and external indices, professional experience was found to be significant in multivariate regression analyses. *Conclusions*. Even though nursing students perceived the severity of the misconduct significantly lower than the experienced nurses, the students demonstrated a greater readiness to blow the whistle, both internally and externally. Recommendations for handling comparable situations are offered.

## 1. Introduction

Whistleblowing is usually defined as the reporting “by organization members (former or current) of illegal, immoral, or illegitimate practices under the control of their employers to persons or organizations that may be able to effect action” [1]. The objective of the whistleblower is to stop the behavior that causes harm to a patient and to prevent such conduct in the future. The reporting can be made to superiors within the employing organization and to authorities outside the organization who are in a position to help, such as regulatory agency with oversight responsibility, journalists, and, if the case merits, even the police.

The act of whistleblowing itself is a thorny dilemma because the individual has to choose between the public good and his or her loyalty to colleagues, supervisors, and/or employer. There is also an imbalance of power involved in the act, and although there are some cases where employers have rewarded whistleblowers for their efforts [2], the typical response of the employee or colleagues is harassment and mistreatment [3, 4].

In the healthcare professions, the dilemma becomes even more complicated because the damaged party is a patient. If a physician, nurse, or other healthcare workers decide to do nothing to stop a colleague's or management's harmful conduct, he or she may be contravening a basic professional commitment to promote and protect patients' health and welfare.

Among the healthcare professions, it is in nursing that the greatest attention has been given to this dilemma [5]. Over the past two and a half decades, researchers of the different fields of nursing have examined its main themes. Since nurses' codes of ethics oblige them to be patient advocates and to take action when a patient's rights or safety is endangered, some studies have concentrated on ethical decision-making [6, 7]. Others have centered on the relation of nursing advocacy with whistleblowing [8–10]. Investigators have also examined the personal and professional risks involved in whistleblowing [11–13]. Other studies have been looking at nurses' knowledge of and beliefs about whistleblowing [14–16], as well as the moral distress caused by the difficult ethical decisions that nurses have to make in light of their divided loyalties at work [17, 18].

In addition to understanding the scope of the ethical dilemma facing nurses, it is also important for both nursing students and practicing nurses with the appropriate tools to address such situations. For nursing students, one way to achieve this is to determine how to integrate whistleblowing into their training so that this does not simply remain part of the “hidden curriculum” where knowledge is not conveyed directly but stems from the professors' assumptions and values, the students' expectations, and the social context in which both teacher and those taught find themselves [19]. For nurses in their professional practice, it is important to give advice on the best way to handle the reporting of a coworker or management misconduct.

In order to design these pedagogical and practical tools, we first need to know what nurses and nursing students are prepared to do when confronted by the harm a patient is suffering at the hands of a colleague or the institution.

The goal of this study was to begin answering this question by comparing nurses willingness to blow the whistle with that of nursing students. To that end, we designed it according to three main parameters. Firstly, we wanted to know if nurses and nursing students were willing to take action to stop misconduct in the workplace in order to protect a patient's interest and how strong their willingness was. Secondly, we wanted to explore to whom they would be willing to report the misconduct, to authorities within the organization and/or to an external authority with the power to intervene and stop the wrongdoing. Finally, we examined whether there were differences between the nursing students and the nurses in their readiness to disclose wrongdoing if the misconduct and harm stemmed from a colleague or from management. The objective was to explore how nurses and students understood their obligation to report the misconduct of a coworker to protect a patient.

## 2. Methods

### 2.1. Sample and Procedure

The convenience sample was comprised of 165 participants divided into two groups: one group of 82 undergraduate nursing students from two nursing schools (one in the central region of Israel and the other in the southern part of the country) and a second group of 83 nurses working in four medical centers in the central and southern regions of Israel. The main distinction between the two groups was professional experience. While the students had no field experience, the nurses had 13.1 years of experience on average (*M* = 13.1; SD = 8.54; range: 1–33 years).

The questionnaires were administered to prospective students in a mandatory course at the end of the second semester. The questionnaires were administered to the nurses at the medical centers during work hours. The distribution and presentation of the questionnaire were identical for all respondents and were done by an experienced research assistant. All prospective responders were informed that the questionnaire was a part of a survey on ethics, that the gathered data would be used only for research purposes, and that participation was completely voluntary and anonymous. After the responders filled out the questionnaires, they were collected by the research assistants, who put them in a sealed envelope and delivered them to the researchers. The administration of the questionnaire lasted for about 10 to 15 minutes. The study was approved by the local ethics committee.

#### 2.1.1. Instrument

The questionnaire was comprised of multiple-choice questions pertaining to sociodemographic information and two vignettes that described ethical dilemmas in which the respondent had to choose between responsibility to the patient and loyalty to a colleague or superior that were likely to arise in the workplace. The sociodemographic section gathered information about age, gender, marital status, and country of origin. The nurses also provided information regarding years of professional experience.

The questionnaire presented two vignettes describing situations in which nurses were required to make a decision involving whistleblowing. Both vignettes described ethical dilemmas in which the nurse had to choose between responsibility to the patient and loyalty to a colleague or a supervisor.

The vignettes were designed according to characteristics specific to acts of whistleblowing. Most accounts of such cases reveal similar methods of action. In general, the process of whistleblowing is a gradual one. First, there is an internal disclosure; that is, the whistleblower approaches a superior or another party who is higher up in the organization's hierarchy in order to put a stop to the conduct that is detrimental to the public or a third party. Afterwards, if this step is insufficient to bring about the desired result, the whistleblower makes an external disclosure to an outside party, such as the press or the police. Organizations that encourage whistleblowing and protect whistleblowers, as well as researchers who study this subject, recommend this method for both strategic and ethical reasons, since it enables the employee to remain loyal to the organization and, at the same time, to strive to put an end to the wrongdoing [20, 21]. An internal disclosure is likely to put a stop to the act and consequently prevent an external disclosure, which may be detrimental to the organization. An internal disclosure that precedes an external one also demonstrates the whistleblower's loyalty to the organization with which he or she is affiliated and even provides him or her with the moral justification for approaching an external party, should all the internal avenues he or she tried prove unsuccessful [22, 23].

The case stories were first presented to five students in order to receive their preliminary responses. These responses were then used to finalize the formulation of the questionnaire. For each case story, there were five questions: question (1) asked the respondent to rate the gravity of the misconduct, questions (2) and (3) dealt with internal whistleblowing, and questions (4) and (5) dealt with external whistleblowing. The first question was rated on a scale of 1 (“not serious at all”) to 7 (“very serious”). The rating of answers to the other questions was on a scale of 1 (“not likely at all”) to 4 (“very likely”). In order to examine the differences between the two types of whistleblowing, questions (2) and (3) were summed into one index, which represents internal whistleblowing, and questions (4) and (5) into one index representing external whistleblowing. The two vignettes were presented as follows.

*Vignette 1*. Protecting the Patient's Interest versus Being Loyal to a Colleague.

You are a nurse in a geriatric center. A colleague submitted his candidacy for a supervisor's job and was selected for the job. You know that the job requires either a master's degree or several years of relevant work experience. You also know that the colleague used a forged degree to get the job and that he does not have the necessary managerial experience—a fact that could harm those cared for by the geriatric center.

1. How grave do you consider your colleague's behavior?
2. How likely is it that you will talk to your colleague and try to persuade him to admit his true level of education and his lack of relevant work experience to his superiors?
3. If you decide not to talk to your colleague, or if you have talked to him about the matter and not succeeded in getting him to admit his true level of training, how likely is it that you will go to someone in the center who has the power to intervene, such as the personnel manager or the center's director?
4. If you do not approach anyone in the center, or if you do talk to someone and he or she does nothing to intervene, how likely is it that you will turn to the Nurses' Association, an external body?
5. If you decide not to talk to the Nurses' Association, or if you do talk to them and they do nothing, how likely is it that you will report the matter to the media?

The internal reliability of the questionnaire (questions (1) to (5)) was adequate (*α* = 0.78). The correlations for the two questions measuring internal whistleblowing and for the two questions measuring external whistleblowing were (*r* = 0.49,  *r* = 0.58), respectively. These high correlations suggest that each of the two questions representing the internal and external whistleblowing indices relates to the same concept.

*Vignette 2*. Protecting the Patient's Interests versus Being Loyal to Management.

You are a nurse in the children's section of a center for victims of violence. It has recently come to your attention that the director of the section intends to use the money budgeted for buying equipment for a play corner to buy luxury fittings for her own office. You have significant reason to believe that not setting up the play corner will significantly delay the recovery of the children cared for by the center.

1. How serious do you consider the director's behavior?
2. How likely is it that you will try to persuade the director not to use the money for her office fittings and to set up the play corner instead?
3. If you decide not to talk to the director, or if you have talked to her and have not been able to change her mind, how likely is it that you will report the director's intentions to someone at the center who has the power to intervene, such as the center's director or the budget director?
4. If you do not refer the matter to an authority at the center, or if you do and he or she does not intervene in the section's director decision, how likely is it that you will turn to the Nurses' Association, an external authority?
5. If you decide not to report the matter to the Nurses' Association, or if you do talk to them and they do nothing, how likely is it that you will report the matter to the media?

The internal reliability of the questionnaire (questions (1) to (5)) was moderate to high (*α* = 0.75). The correlations for the two questions measuring internal whistleblowing and for the two questions measuring external whistleblowing were *r* = 0.47 and *r* = 0.65, respectively. These high correlations suggest that each of the two questions representing the internal and external whistleblowing indices relates to the same concept.

### 2.2. Statistical Analysis

Mean differences between groups were assessed using Student's *t*-test. The relative contribution of different variables to the explanation of the severity of the misconduct and the indices of internal and external whistleblowing was assessed using multiple linear regression analysis. The internal reliability of the questions comprising the case stories was assessed using Cronbach's alpha coefficient. Significance level was set at *P* < 0.05 for all analyses. The data were analyzed with SPSS statistical software, PC version 17.0.

## 3. Results

The response rate was high for both groups (76%, 83/109 for the nurses, and 83%, 82/100 for the undergraduate nursing students).

No statistically significant differences were found between the two groups for the demographic characteristic of gender. However, statistically significant differences were found with regard to age, marital status, and country of birth. Participants in the nurses group were older than the participants in the students group, and a higher percent of them were married and were born outside of Israel. A comparison between the sociodemographic characteristics of both groups is presented in Table 1.

Because significant differences between the two groups were found for the sociodemographic characteristics of age, marital status, and country of birth, these variables as well as the variable of professional field experience (inexperienced students/experienced nurses) were submitted to regression analysis in order to establish each variable's unique contribution to the variance of the assessed indices. Regression analysis was conducted for the explanation of the perceived severity of the misconduct and the internal and external whistleblowing indices in both case stories (Tables 2 and 3).

None of the studied variables were found to be significant predictor of the severity of the misconduct for both case stories.

For each of the examined internal and external indices, professional experience was found to be statistically significant. In other words, for both case stories, the nursing students, who did not have professional experience, had a greater tendency toward internal and external whistleblowing in order to change the situation in comparison with the experienced nurses. It should be noted that age was also found to be a statistically significant predictor of the internal and external whistleblowing indices for both case stories. The relative contribution of age to the explanation of the whistleblowing indices was found to be similar to that of experience.

A comparison between the mean scores of the nursing students and the experienced nurses regarding the severity of the misconduct and the indices of internal and external whistleblowing for both vignettes is presented in Table 4.

Both groups rated the two vignettes as very serious. The nursing students perceived the severity of the misconduct of the colleague and the manager significantly lower compared to the nurses. No significant statistical differences were found between the two groups regarding the internal and external whistleblowing indices for both vignettes. Yet, a tendency was found—the students ranked the two whistleblowing indices higher than the nurses. In other words, the students reported that they were more likely to approach parties within the organization and external to it in order to change the situation compared to the nurses.

## 4. Discussion

The study findings indicate that both the nurses and the nursing students viewed acts that are detrimental to the patient in a very serious light. In situations such as these, they reported a willingness to act in cases where both a colleague and management were involved. There was a significant difference between the two groups, however. Even though nursing students perceived the severity of the misconduct significantly lower compared to the nurses, the students reported a greater readiness to blow the whistle, both internally and externally.

The fact that the students were more willing to act internally and externally than the professional nurses might stem from a lack of awareness of the risks that such an intervention could mean for them, other patients, and/or the organization concerned. It might also reveal a lack of prudence. Case studies in the literature, including those on other health and care professions, clearly indicate that the price paid by the whistleblowers in some cases could be very high [24]. Due to their experience, nurses might be aware of such facts, whereas students may not. But it could also be the case that it is the socialization process that nurses go through in the working place. Also fear of management's power to retaliate might play a part in his or her decision. These are questions that should be pursued in future studies.

Such studies are important in both pedagogical and practical terms. The idea is that both student and professional nurses would be equipped with the necessary tools to confront such problematic situations. For students, it would be a matter of integrating the subject of whistleblowing into the nursing curriculum. The aims of this pursuit would be to broaden the basis of ethical education, provide an additional anchor for the principle of the patient's best interests, and furnish prospective nurses with the tools to handle similar situations in their future practice. We consequently recommend that, in addition to studying the ethical aspects of reporting wrongdoing, researchers and practitioners also consider whistleblowing as a tool for advocacy and social intervention [25–27]. At the practical level, we recommend that nurses should receive information through such channels as their professional association, with regard to the existing legislation protecting whistleblowers in their country and on the governmental and/or nongovernmental organizations established to advise and support potential whistleblowers, thereby making it safer to take action to stop workplace wrongdoing.

Finally, the question of* how* to blow the whistle is no less important than that of* whether* to blow it or not. Many organizations have created internal channels for reporting misconduct, and our recommendation is to use them when necessary. Such avenues have the great advantage of avoiding the damage that public exposure might bring to the institution and to the whistleblower him/herself. At the same the time, they help strengthen an individual's professional ethics and values.

One limitation of the study needs to be acknowledged. It reflects the study participants' self-expectations of how likely they are to report the portrayed wrongdoing rather than their actual reporting. The self-assessment of the participants does not necessarily indicate what the respondents will actually do should they encounter the misconduct described in the vignettes. The literature on attitudes repeatedly points to large disparities between an individual's beliefs and his or her behavior [28]. It is therefore not possible to predict precisely what the respondents would do when they actually face misconduct. It could be that when faced with a real situation, some respondents who declared themselves unlikely to report misconduct will decide to take action; and some of those who declared themselves likely to report it will not actually go ahead.

## Figures and Tables

**Table 1:** A comparison between the sociodemographic characteristics of the undergraduate nursing students group and the nurses group.

Variable	Undergraduate nursing students (*n* = 82)		Nurses (*n* = 83)		*t*/*χ* ^2^
	*N* (%)	Mean (SD)	*N* (%)	Mean (SD)	
Age (years)	23.53	2.02	38.09	8.75	14.68∗∗
Gender					
Men	14 (17.1)		8 (9.6)		3.43
Women	68 (82.9)		75 (90.4)		
Marital status					
Married	9 (11.0)		60 (72.3)		63.7∗∗
Other	73 (89.0)		23 (27.7)		
Country of birth					
Israel	49 (59.8)		37 (44.6)		3.85∗
Other	33 (40.2)		46 (55.4)		

^∗^ *P* < 0.05; ^∗∗^ *P* < 0.001.

**Table 2:** Linear regression: predictors for the explanation of the severity of the misconduct and the internal and external whistleblowing—ethical dilemma involving a colleague at work (*n* = 165).

	Professional experience^1^	Age	Marital status^2^	Country of birth^3^
	*Β*	*Β*	*β*	*β*
Severity of the colleague's misconduct	−0.20	−0.03	−0.04	−0.15

Internal whistleblowing index: approach the colleague or authority figure in the organization	0.34∗∗	0.31∗	0.08	0.02

External whistleblowing index: approach the Nurses' Association or the media	0.35∗∗	0.37∗∗	0.07	0.03

^*^ *P* < 0.05; ^**^ *P* < 0.01; ^1^0 = experienced nurses; 1 = inexperienced nursing students; ^2^0 = married; 1 = other; ^3^0 = Israel; 1 = other.

**Table 3:** Linear regression: predictors for the explanation of the severity of the misconduct and the internal and external whistleblowing—ethical dilemma involving a manager at work (*n* = 165).

	Professional experience^1^	Age	Marital status^2^	Country of birth^3^
	*Β*	*β*	*β*	*β*
Severity of the manager's misconduct	−0.08	0.06	−0.08	−0.14

Internal whistleblowing index: approach the manager or authority figure in the organization	0.25∗	0.28∗	0.14	0.03

External whistleblowing index: approach the Nurses' Association or the media	0.32∗∗	0.36∗∗	0.17	0.11

^*^ *P* < 0.05; ^**^ *P* < 0.01; ^1^0 = experienced nurses; 1 = nursing students (inexperienced); ^2^0 = married; 1 = other; ^3^0 = Israel; 1 = other.

**Table 4:** A comparison between the severity of the misconduct and the indices of internal and external whistleblowing for the undergraduate nursing students and the nurses, in the two vignettes.

	Possible range	Undergraduate nursing students (*n* = 82)		Nurses (*n* = 83)		*t*
		Mean	SD	Mean	SD	
Vignette 1-colleague						
Severity of the misconduct	1–7	6.59	0.90	6.86	0.47	2.41∗
Internal whistleblowing	2–8	6.31	1.39	5.95	1.44	−1.61
External whistleblowing	2–8	4.38	1.59	4.11	1.62	−1.08
Vignette 2-manager						
Severity of the misconduct	1–7	6.74	0.56	6.89	0.39	1.95∗
Internal whistleblowing	2–8	6.74	1.18	6.55	1.33	−0.99
External whistleblowing	2–8	5.21	1.75	4.89	1.81	−1.14

^*^ *P* < 0.05.
